# Radiofrequency ablation with sine and square electrical waveforms to enhance ablation range

**DOI:** 10.3389/fbioe.2024.1450331

**Published:** 2024-08-21

**Authors:** Dong-Sung Won, Jinsu An, Ji Won Kim, Yubeen Park, Sang Soo Lee, Hyung-Sik Kim, Jung-Hoon Park

**Affiliations:** ^1^ Biomedical Engineering Research Center, Asan Institute for Life Sciences, Asan Medical Center, Seoul, Republic of Korea; ^2^ Department of Convergence Medicine, Asan Medical Center, University of Ulsan College of Medicine, Seoul, Republic of Korea; ^3^ Department of Biomedical Engineering, School of ICT Convergence Engineering, College of Science and Technology, Konkuk University, Chungju-si, Chungcheongbuk-do, Republic of Korea; ^4^ Department of Gastroenterology, Asan Medical Center, University of Ulsan College of Medicine, Seoul, Republic of Korea; ^5^ Department of Mechatronics Engineering, School of ICT Convergence Engineering, College of Science and Technology, Konkuk University, Chungju-si, Chungcheongbuk-do, Republic of Korea

**Keywords:** radiofrequency ablation, local heat treatment, electrical waveforms, ablation ranges, tumor ablation

## Abstract

Radiofrequency ablation (RFA) is a local treatment modality for primary liver cancers. Although various input parameters of the RF generator have been adjusted to improve the ablation ranges, the limited ablation ranges remain an obstacle to RFA. This study aimed to compare the ablation ranges and efficacy of sine and square electrical waveforms in a mouse tumor model. An RF generator with an adjustable electrical waveform was developed, and its ablation range in the porcine liver was compared. For all RF parameters, the square electrical waveform ablation range was greater than that of the sine electrical waveform (all *p* < 0.001) in the porcine liver. The 45 BALB/c nude mice were used to evaluate the efficacy of the two electrical waveforms after the RFA. The mean tumor volume in the square group was significantly lower than that in the sine group (*p* < 0.001), indicating a higher survival rate (60%). The cellular coagulative necrosis, inflammatory cell infiltration, heat shock proteins, cellular necrosis, and tumor necrosis were significantly greater in square electrical waveform than in sine electrical waveform (all; *p* < 0.05). RFA with square electrical waveforms has therapeutic potential for tumor management with an enhanced ablation range.

## Introduction

The incidence of primary liver cancer including hepatocellular carcinoma (HCC) and intrahepatic cholangiocarcinoma (ICC) is steadily increasing and is an important cancer-related mortality source of worldwide (Massarweh and El-Serag, 2017). Surgical resection of primary liver cancer is a standard therapeutic option with preservation of adequate function of the residual livers (Ambrogi et al., 2006; Orcutt and Anaya, 2018). Although the safety of surgical resection has improved in recent years, it is not always warranted for patients with poor functioning, unfit to withstand anesthesia, and unresectable malignancies (Marin-Hargreaves et al., 2003; Shah et al., 2013). Radiofrequency ablation (RFA) has been demonstrated to be safe and promising for primary and secondary liver cancer under 3 cm in size resulting from tumor necrosis by delivering heat agitation (Cho et al., 2009; Steel et al., 2011). Nevertheless, RFA has a limitation in that the high tumor recurrence rate in relatively large tumors over 3 cm has been reported due to insufficient coagulative necrosis, and incomplete ablation defect margin (Lee et al., 2004; Schraml et al., 2008; Wang et al., 2013; Xiang et al., 2020). Recently, a combination therapy to increase therapeutic outcomes and decrease recurrence rate was actively investigated such as RFA with neoadjuvant transarterial chemoembolization, chemical ablation, and chemotherapy (Shah et al., 2013; Kim et al., 2019). However, the aforementioned methods may correlate with increased patient inconvenient such as the necessity of additional procedures, increasing procedure-related side effects, and low-cost effectiveness (Pleguezuelo et al., 2008; Lencioni et al., 2004).

To enhance ablation ranges, several investigations have been conducted to adjust the RF generator’s input parameter to maximize the extent of coagulative necrosis such as amplitude variation, frequency, and waveform (Goldberg et al., 1999; Lim et al., 2010; Sydorets et al., 2016). Lim et al. (2010) reported comprehensive information on the relationship between electrical waveforms and the thermal response of the tissue to determine the effect of the input waveform pattern to increase the extent of tumor necrosis. The electrical square waveform is characterized by its rectangular shape with sharp rising and falling (Novickij et al., 2022). Therefore, the square electrical waveform, which contains many harmonics, can generate more heat in the tissue (Sonerud et al., 2009; Li et al., 2020). Theoretically, the energy of square electrical waveform is delivered a constant overtime because calculation of its absolute value (Bird, 2017). The temperature ripple could be small in a temperature-controlled environment. Due to these characteristics, it was assumed that square electrical waveform could generate more heat and maintain small temperature fluctuation than sine electrical waveform. Additionally, several studies have demonstrated that square electrical waveforms with increased conductivity are more efficient at transferring energy than triangular and sine electrical waveforms (Kotnik et al., 2003; Miklavcic and Towhidi, 2010). However, the previous studies performed only in waveform amplitude or *ex-vivo/in-vitro* without reproducible model for clinical practice. The newly developed RF generator can be selectively ablated using sine or square electrical waveform. Therefore, this study aimed to compare the ablation ranges and efficacy of sine and square electrical waveforms in porcine liver and tumor mouse models.

## Materials and methods

### Radiofrequency generator and electrode

**FIGURE 1:**
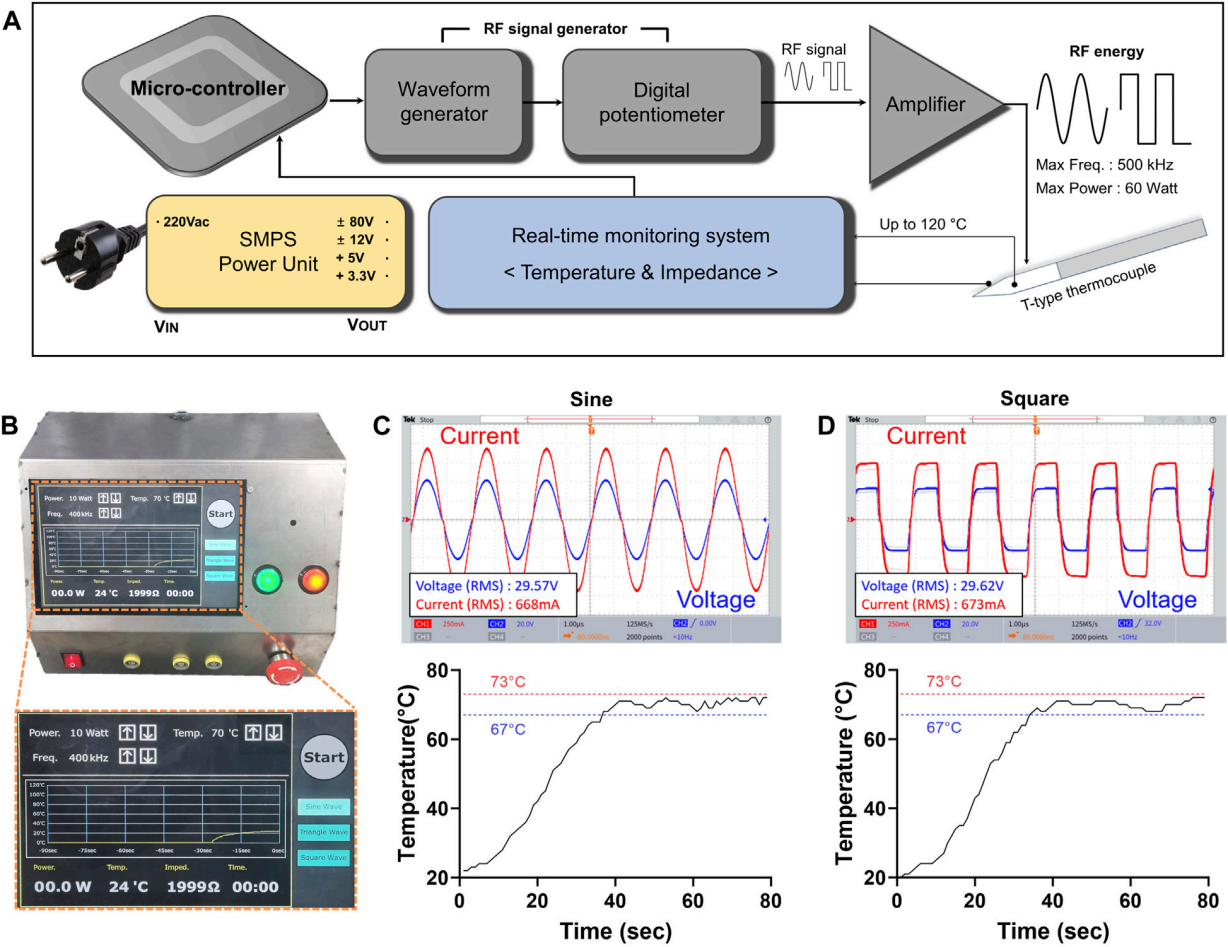
RF generator with adjustable sine or square electrical waveforms. **(A)** The block diagram of the developed RF generator. The RF generator is controlled by a microcontroller and consisted of waveform generator, a real-time monitoring system. The real-time monitoring system measures temperature and impedance. The RF energy is amplified to a maximum output of 60 W through the amplifier. **(B)** Photographs of the generator and magnified the display system. Characterization including output voltage, current waveform, and temperature profile of each **(C)** sine and **(D)** square electrical waveforms at 20 W and 400 kHz RF parameters.

In this study, a newly developed RF generator was used to perform RFA procedure. The waveform, output power, operation time, and temperature were adjustable within a specific range (Supplementary Table S1). Real-time measurements of temperature and impedance were possible during the procedure. The developed RF generator is shown in Figures 1A, B, as well as in Supplementary Figure S1. The RF generator is controlled by a microcontroller, ATMEGA128A1 (Microchip Technology, Inc., Chandler, AZ, United States), which manages the entire system. The frequency and power are controlled through a programmable waveform generator, AD5932 (Analog Devices, Inc., Wilmington, MA, United States), and a digital potentiometer, AD8402 (Analog Devices, Inc.). The waveform can be selected between sine and square electrical wave. The RF signal generated by the waveform generator is amplified through a high-voltage power-operational amplifier, PB64DP (Apex Microtechnology, Tucson, AZ, United States), and used as the output of the RF generator. The maximum frequency of the RF generator is 500 kHz, and the maximum output power is 60 W. For real-time temperature monitoring, an instrumentation amplifier, AD620 (Analog Devices, Inc.). A T-type thermocouple was used, enabling temperature measurements up to 120°C. The temperature control can be set by the user in 5°C increments from 50°C to 100°C. A digital temperature data logger (MV1000, Yokogawa Electric Co., Japan) was used to calibrate the temperature. Impedance measurements employed an analog front-end integrated circuit (ADS1292; Texas Instruments Inc., Dallas, TX, United States). It is capable of impedance measurements within the range of 10 Ω to 1.5 kΩ and has been calibrated using a low-frequency (LF) impedance analyzer (4192A; Hewlett-Packard Co., Palo Alto, CA, United States). This facilitates the assessment of the contact status between tissue and electrode, as well as monitor the impedance changes before and after applying RF energy. A commercially available monopolar RF electrode (VIVA II RF Electrode; Taewoong Medical Co., Kimpo, Korea) was used for RFA in both *ex-vivo* and *in-vivo* studies. The electrode, which included a T-type thermocouple, was 18G, and the exposed 0.7 cm of its tip was fixed for reproducibility.

### Radiofrequency generator evaluation

The RF energy and temperature were measured to verify the operation of the RF generator. Using a power resistor with the same impedance as the mouse cancer model, specifically 42 Ω, RF power outputs of 5, 10, 15, and 20 W were applied for sine or square electrical waveform. The impedance value of the mouse cancer model was obtained in advance using an LF impedance analyzer (4192A; Hewlett-Packard Co., Palo Alto, CA, United States). The output voltage and current of the RF energy were measured using an oscilloscope (TBS200B; Tektronix Inc., Beaverton, OR, United States) with a high-voltage differential probe (P5210A; Tektronix Inc.) and a current amplifier (TCPA300; Tektronix Inc.). The output wattage was measured by multiplying the output voltage and current, and the output error was calibrated to be less than 10%. The temperature control profile was evaluated by attaching a T-type thermocouple and a digital temperature data logger (MV1000) to the power resistor. The same temperature control profile, set to 70°C, was applied for both electrical waveforms..

### Ablation ranges of sine and square electrical waveforms in the porcine liver

The ablation range was investigated using a porcine liver to confirm the sine and square electrical waveforms according to changes in RF-related parameters. Porcine livers were sectioned into cuboidal specimens to evaluate the ablation range using sine and square electrical waveforms. The liver specimen was placed on a ground pad and a needle-type electrode was penetrated at the middle portion of the cuboid liver. RFA was conducted with fixed parameters at 400 kHz, and 70°C for 300 s by crossing four RF powers (5, 10, 15, and 20 W) and sine or square electrical waveform. The RFA-treated liver was cut along the long axis of the needle-type electrode. All RFA-treated livers were photographed and the ablation range was measured using Vernier calipers. The ablation length was measured as the maximum diameter ablated along the axis of the needle-type electrode, and the ablation depth was measured as the maximum diameter ablated along the vertical axis of the needle-type electrode. The ablation zone was calculated using the formula: Volume (V) = π/6 × depth^2^ × length (Zhang et al., 2018). All studies were repeated 10 times to determine statistical reproducibility.

### Cell culture

The colon adenocarcinoma cell line (CT-26, Korean Cell Line Bank, Seoul, Korea) was cultured in RPMI-1640 (R8758; Sigma Aldrich, St. Louis, MO, United States) supplemented with 5% fetal bovine serum (SH30919.03; GE Healthcare Life Sciences, Logan, UT, United States) and 1% penicillin−streptomycin (17-745E; Lonza Bioscience, Walkersville, MD, United States). Cells were incubated at 37°C in a humidified atmosphere containing 5% CO_2_ in an incubator and sub-cultured was performed every 48 h in the same environment. Cell suspensions were prepared via enzymatic treatment with trypsin–EDTA (Life Technologies, Gaithersburg, MD, United States). Cell viability >95% was checked by trypan blue staining before the implantation procedure (Supplementary Figure S2).

### Mice tumor model

The animal study was approved by the Institutional Animal Care and Use Committee (IACUC) of the Asan Institute for Life Sciences (IACUC No. 2023-20-086, Seoul, Korea) and the study was conducted in compliance with the ARRIVE guidelines. A total of 45 8-weeks-old male BALB/c nude mice (weighing 20–23.5 g; JA Bio, Suwon, Korea) were acclimatized for 7 days before injecting colon cancer cell lines. Mice were anesthetized with 2.0% isoflurane and placed in a prone position on the heating pad. CT-26 cells (1 × 10^5^ cells) were subcutaneously injected into the right or left flank to create a mouse tumor model. Each mouse was weighed every 2 days until sacrifice. Additionally, all animals were monitored every 2 days to measure tumor growth using a caliper, and tumor volume was calculated using the following formula: length × width^2^ × 0.5. Mice with tumor volumes reaching 1,000 mm^3^ within 14 d of tumor cell implantation were included in the study.

### Animal study design

**FIGURE 2:**
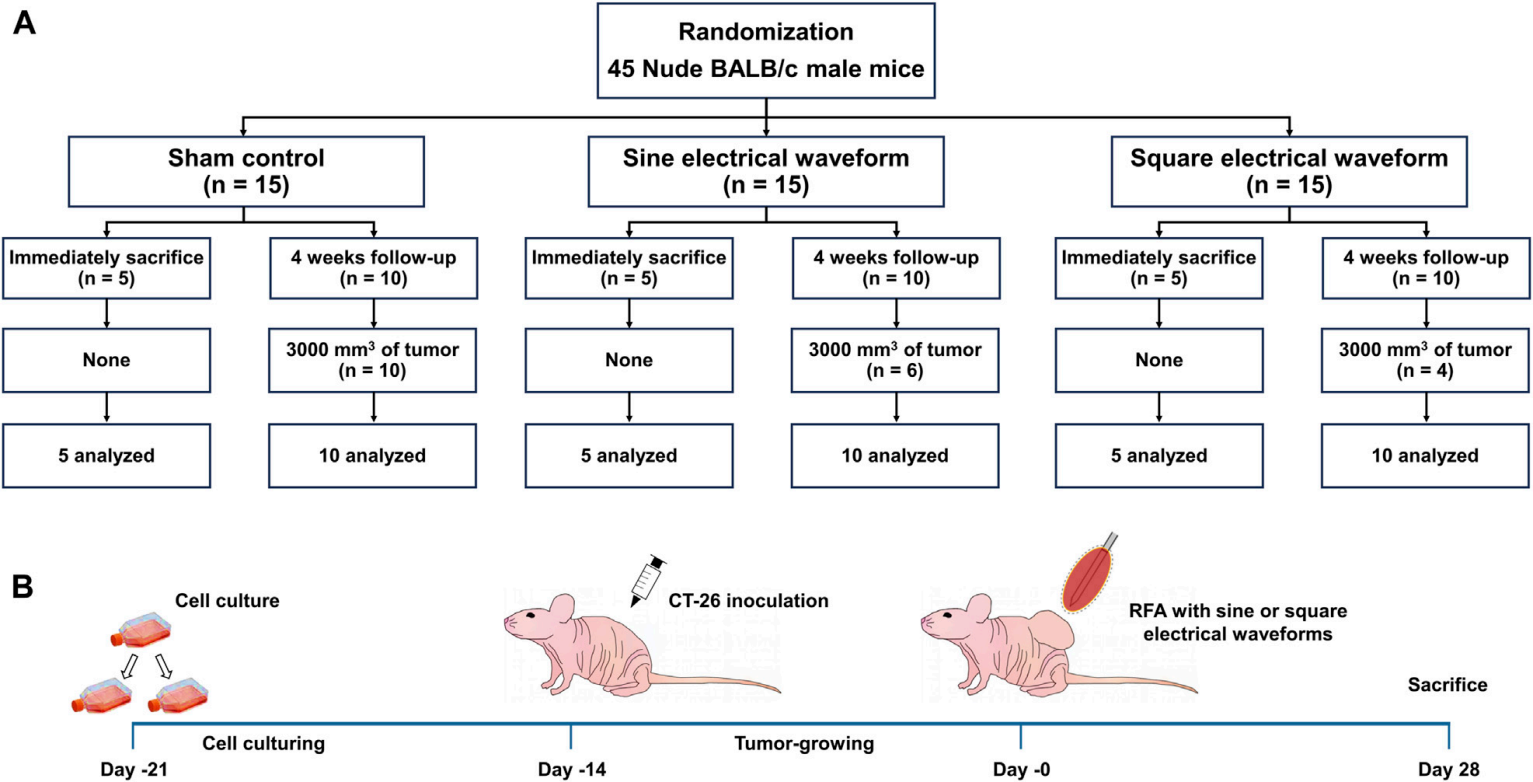
Overall *In-vivo* study scheme in the mice tumor model. **(A)** Flow chart of the *in-vivo* study design and follow-up. **(B)** Schematic representation of the experimental design in the mice tumor model to apply the RFA treatment with sine or square electrical waveforms.

The average tumor volume of 1,000 mm^3^ was achieved 14 days after tumor cell implantation. A total of 45 mice were then randomly assigned to three groups: the sham control group (n = 15) received electrode penetration without RFA, the sine group (n = 15) received monopolar RFA with a sine electrical waveform, and the square group (n = 15) received monopolar RFA with a square electrical waveform. The 5 of 15 mice in each group were sacrificed immediately after the RFA procedure to evaluate the efficacy of the two RFA electrical waveforms. The remaining 10 mice in each group were sacrificed at the study endpoint by inhalation of pure carbon dioxide. The mice were sacrificed 4 weeks after the RFA procedure; however, any mouse whose with a residual tumor volume reached 3,000 mm^3^ was considered dead (Figure 2). All animals were housed on a 12-h light/dark cycle at proper environmental temperature (24°C ± 1°C) and humidity (55% ± 10%). Three mice were housed per cage, with free access to food and water.

### Radiofrequency ablation with sine or square electrical waveforms in a mouse tumor model

The mice were anesthetized with 2.0% isoflurane in the lateral decubitus position and placed on a metallic grounding pad with a contact gel. Before the procedure, ultrasonography was performed to identify non-necrotic solid tumors. An 18G needle-type electrode was inserted into the tumor center under ultrasonographic guidance. The RFA parameters were set to 300 s at a power of 20 W and 400 kHz based on an *ex-vivo* study. Here, we chose the temperature of 70°C as the ablation parameter because the temperature point was the highest tolerated by mice during RFA treatment (Jiang et al., 2022). A thermal camera (FLIR A400; Teledyne FLIR, Wilsonville, OR, United States) was used to obtain temperature changes and thermal images during the procedure. Here, we chose the temperature of 70°C as the ablation parameter because the temperature point was the highest tolerated by mice during RFA treatment (Jiang et al., 2022). Ultrasonography was performed immediately after the RFA procedure to confirm tumor tissue changes. A painkiller (Ketorolac; Hana Pharm, Seoul, Korea) was routinely administered for 3 days after the procedure.

### Magnetic resonance imaging

Magnetic resonance imaging (MRI) was performed using a 9.4-T/160-mm animal MRI system (Agilent Technologies, Santa Clara, CA, United States) before and immediately after the RFA procedure and before sacrifice in all enrolled mice. The mice were anesthetized with 2% isoflurane, and the extended extremities were placed on the mouse bed in a prone position. T2-weighted imaging (T2WI) was performed to confirm changes in the tumor environment. RF excitation and signal detection were accomplished using a 40 mm millipede volume coil. The imaging protocol included a T2WI [(TR = 4000 ms; TE = 31.78 ms; slice number = 25; slice thickness = 0.80 mm; field of view = 40 × 40 mm; and matrix = 256 × 256 (no gap)]. All MRI data were reconstructed from the axial section of the tumor. All tumor volumes were measured on MRI images on a PACS workstation including length, width, depth. Length was defined as the maximum diameter of the tumor, width as the maximum diameter vertical to the length of the axial section, and depth as the maximum diameter of the coronal section. The tumor volume was calculated by using the formula: 4/3π (length × width × depth/2) (Wang et al., 2020). The calculated tumor volume has described the change in volume as a percentage based on before the RFA procedure.

### Histological analysis

The extracted tumor tissues were fixed in 10% neutral buffered formalin for 24 h and embedded in paraffin. Tumor tissues were sectioned transversely for microscopic examination. The slides were stained with hematoxylin and eosin (H&E) to evaluate the cellular changes in the inflammatory reaction after RFA procedure, and Masson’s trichrome (MT) to confirm the fibrotic changes in the RFA-treated tumor tissues. Histological evaluation using H&E and MT slides included the determination of the degree of cellular coagulative necrosis, degree of inflammatory cell infiltration, degree of fibrotic changes, and degree of collagen deposition. The overall degree or grade of coagulative necrosis was determined subjectively based on the severity and distribution of the necrotic lesions and the number of ablated areas affected (graded as: 1, mild; 2, mild to moderate; 3, moderate to marked; 4, severe to diffuse) (Raczynski et al., 2012). The degree of inflammatory cell infiltration was subjectively determined according to the inflammatory cell distribution and density (graded as 1, mild; 2, mild to moderate; 3, moderate; 4, moderate to severe; and 5, severe). The degree of collagen deposition was subjectively determined using MT-stained sections, where 1, 2, 3, 4, and 5 indicated mild, mild-to-moderate, moderate, moderate-to-severe, and severe, respectively.

### Immunohistochemistry

Immunohistochemistry (IHC) was performed on paraffin-embedded sections using heat shock protein 70 (HSP 70; LifeSpan BioSciences Inc., Seattle, WA, United States), terminal deoxynucleotidyl transferase-mediated dUTP nick and labeling (TUNEL; Sigma Aldrich, St. Louis, MO, United States), and tumor necrosis factor-alpha (TNF-α; Abcam, Cambridge, England) primary antibodies to confirm the ablation zone and cellular necrosis after RFA treatment. The extents of HSP 70, TUNEL, and TNF-α -positive deposition were subjectively determined (1, mild; 2, mild-to-moderate; 3, moderate; 4, moderate-to-severe; and 5, severe). All histological analyses were performed using a digital slide scanner (Panoramic 250 FLASH III; 3D Histech Ltd., Budapest, Hungary) and a digital microscope viewer (CaseViewer; 3D Histech). Analyses of the histological findings were based on the consensus of three observers who were blinded to the groups.

### Statistical analysis

Data were expressed as a mean ± standard deviation (SD) and differences between the groups were analyzed using the Student’s t*-test* or one-way ANOVA test, as appropriate. *p*-values < 0.05 were considered statistically significant. The SPSS software (version 27.0, IBM, Armonk, NY, United States) was used to for all statistical analyses.

## Results

### Compatibility between the RF generator and RF electrode

The developed RF generator successfully delivered RF energy to a test resistive load through an electrode. The electrical waveforms of the voltage, current, and temperature were measured under four different output conditions, and are summarized in Supplementary Table S2 and shown in Figures 1C, D. In the sine electrical waveform, the maximum output voltage was 41.8 V, and the current was 944 mA at 20 W and 400 kHz. In the square electrical waveform, the maximum output voltage was 29.62 V, and the current was 673 mA at 20 W and 400 kHz. Temperature was regulated within ±3°C for sine and ±3°C for square electrical waveform. Impedance was measured with an error rate of less than 10% across the entire range of 10 Ω to 1.5 kΩ.

### Ablation ranges of sine and square electrical waveforms in the porcine liver

**FIGURE 3:**
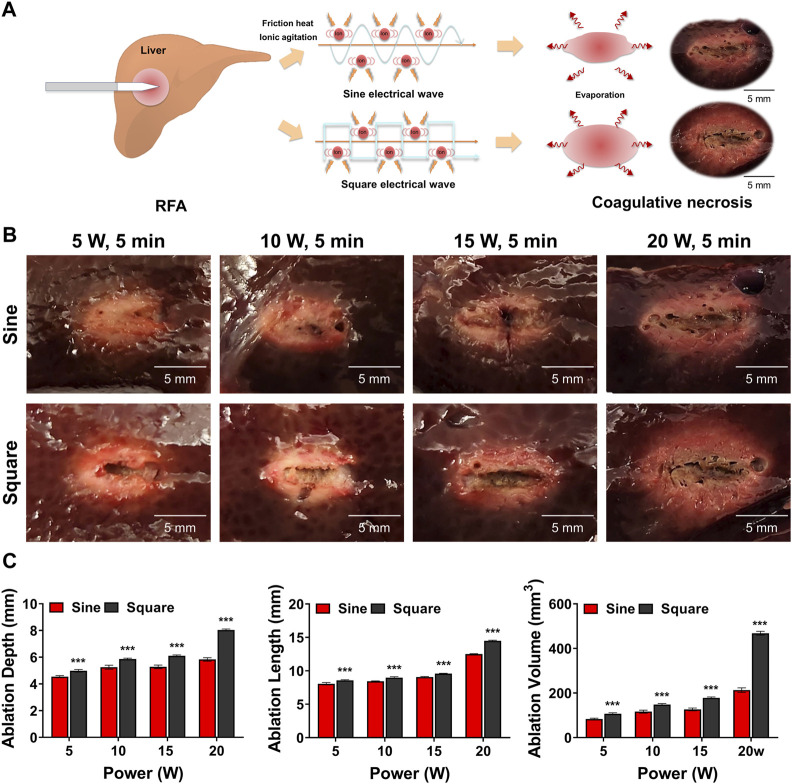
*Ex-vivo* findings after radiofrequency ablation (RFA) in the porcine liver. **(A)** Schematic illustration of RFA with the sine or square electrical waveform in the porcine liver **(B)** Representative photographs of the RF-ablated liver using the sine or square electrical waveform. **(C)** Graphs showing the ablation depth, length, and volume in the RF-ablated liver. Note *** <0.001.

RFA with a sine or square electrical waveform was technically successful in all porcine livers without roll-off. The ablation ranges of the sine and square electrical waveforms are shown in Figure 3A. The ablation ranges in the square group showed a relatively spherical shape and a greater ablation zone than those in the sine group for all RF parameters (Figure 3B). The mean ablation depth, length, and volume were significantly greater in the square group than in the sine group for all parameters (all variables, *p* < 0.001) (Figure 3C).

### Procedural outcomes in a mouse tumor model

**FIGURE 4:**
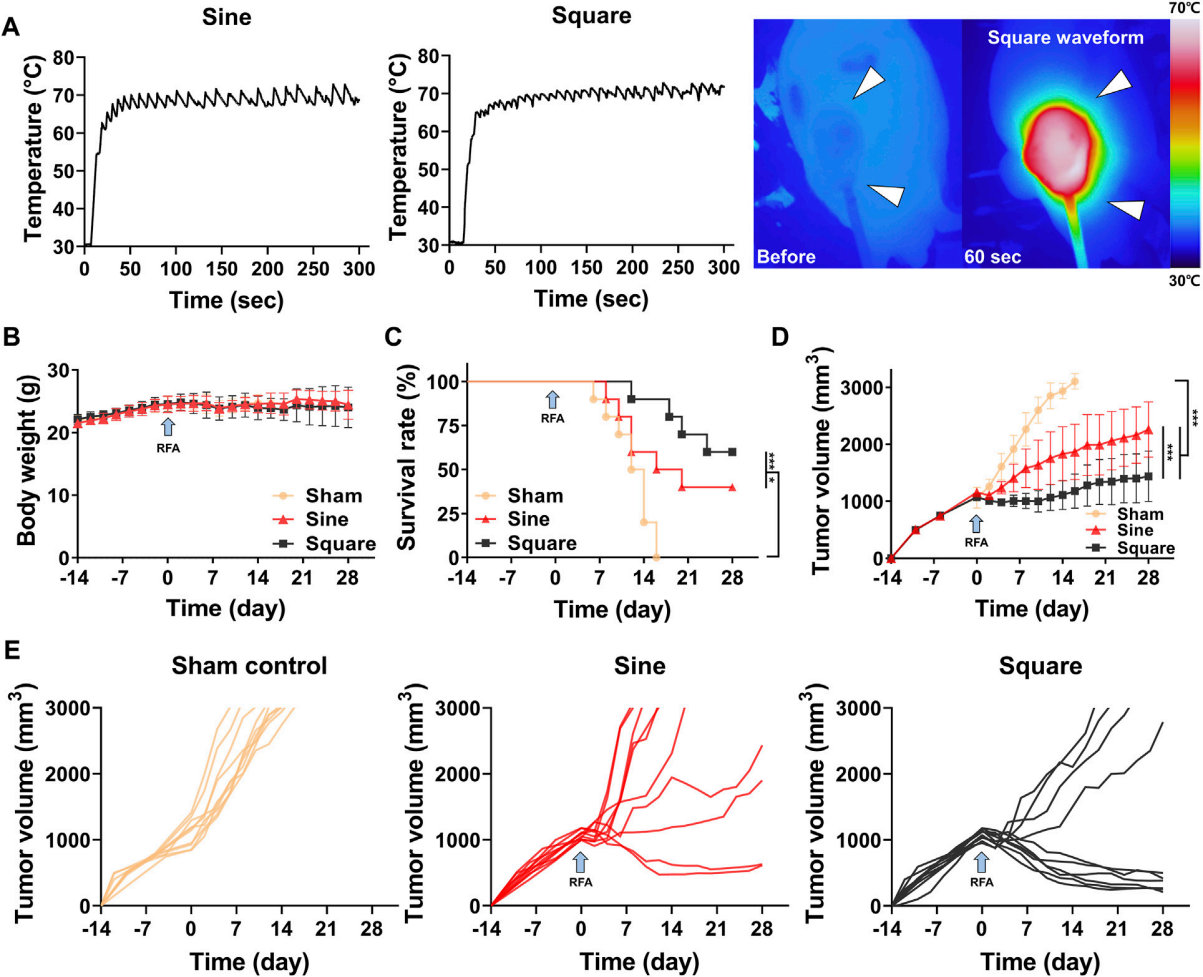
*In-vivo* findings after radiofrequency ablation (RFA) in the mouse tumor model. **(A)** Graphs showing temperature changes during the RFA procedure with sine or square electrical waveforms and thermal images obtained during RFA with the square waveform to the tumor (*arrowheads*). **(B)** Body weight changes after CT-26 cell inoculation in all study groups during the follow-up duration. **(C)** Survival rates of the study groups after with/without RFA. **(D)** Graph showing the mean tumor volume changes after with/without RFA treatment in the study groups. **(E)** Graphs showing the tumor volume changes in each mouse of the sham control, sine, and square groups. Note * <0.05, *** <0.001. RFA, radiofrequency ablation.

The RFA procedure with sine or square electrical waveforms was technically successful in all enrolled mice. Penetration of the needle-type electrode into the middle portion of the tumor tissue was technically successful under ultrasonographic guidance (Supplementary Figure S3). The mean steady-state temperature was 68.95°C ± 1.35°C in the sine group and 70.12°C ± 1.15°C in the square group (Figure 4A). The temperature of sine and square electrical waveform was gradually increased up to 70°C within 50 s and 70 s, respectively. There was no significant difference in body weight between the study groups after RFA with sine or square electrical waveforms (Figure 4B). The residual tumor volume gradually reached 3,000 mm^3^ after tumor cell implantation at 20–30 days (mean, 26.2 days) in 10 of 10 mice (100%) in the sham control group, 22–34 days (mean, 27 days) in 6 of 10 mice (60%) in the sine group, and 26–38 days (mean, 32.5 days) in 4 of 10 mice (40%) in the square group. Twenty of 30 mice (66.67%) with tumor volumes over 3,000 mm^3^ were considered as dead state. Survival rates were higher 60% (6 of 10 mice; *p* < 0.001) in the square group and 40% (4 of 10 mice; *p* < 0.05) higher in the sine group than those in the 0% (0 of 10 mice) sham control group (Figure 4C).

### Tumor volume changes after RFA with sine or square electrical waveforms

The mean tumor volume was significantly different between the groups (*p* < 0.001) (Figure 4D). The tumor volume in the RFA-treated groups decreased for 2 days after RFA owing to water evaporation within the tumor. The tumor volumes in all study groups gradually increased after RFA. However, the mean tumor volumes in the square and sine groups were significantly lower than those in the sham control group (*p* < 0.001). The mean tumor volume in the square group was significantly lower than that in the sine group (*p* < 0.001). Furthermore, the mean tumor volume was significantly lower in the square group (1,443.49 ± 897.89 mm^3^; *p* < 0.001) than in the sine group (2,290.60 ± 1,126.82 mm^3^) at 28 days (Figure 4E).

### Magnetic resonance imaging findings

**FIGURE 5:**
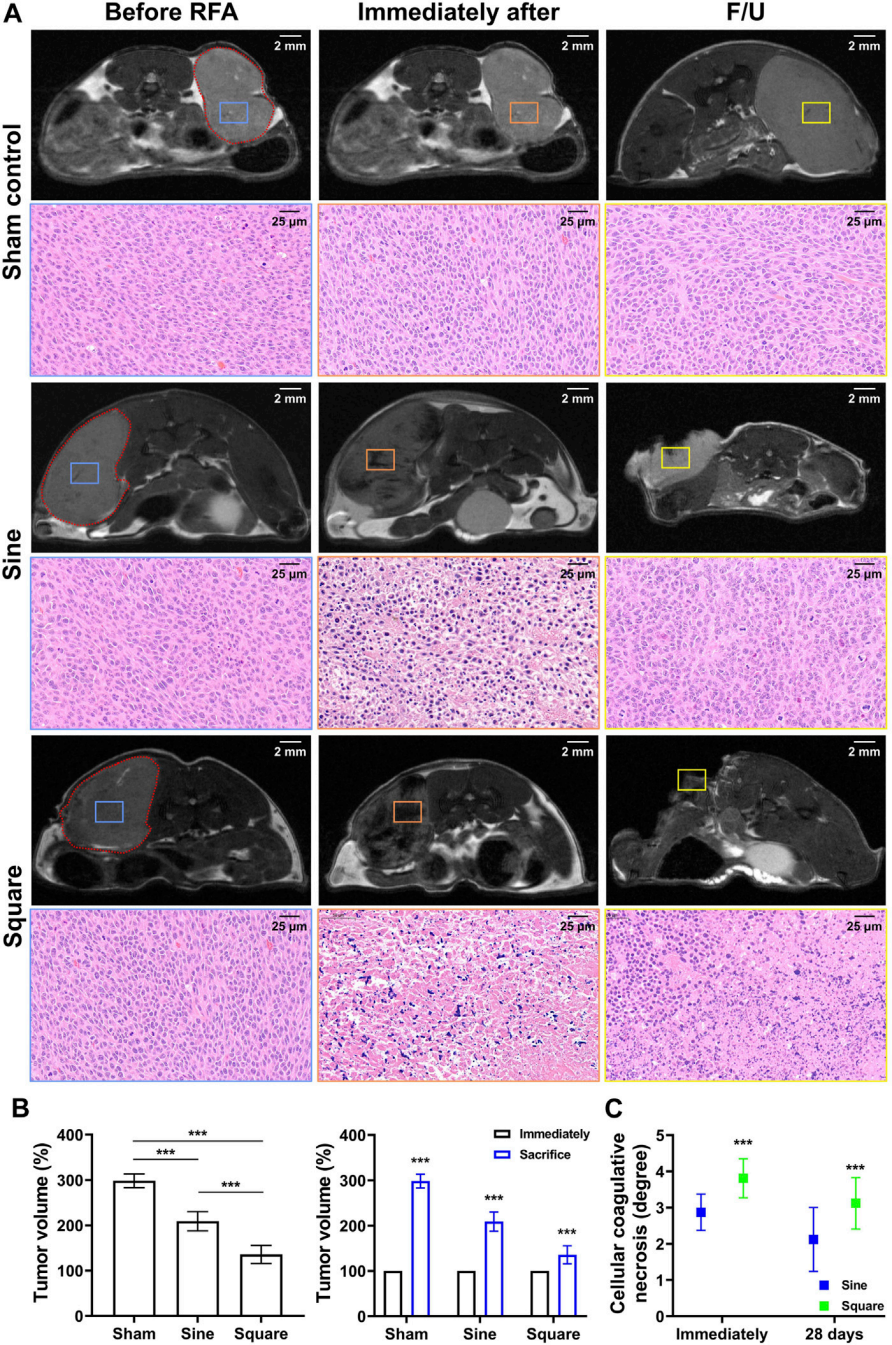
*In-vivo* experimental imaging and histological findings after radiofrequency ablation in the mice tumor model. **(A)** Representative MRI findings immediately after RFA treatment and before sacrifice and histological findings immediately after RFA treatment and at 28 days. The tumor was identified in MRI images (*red dotted line*) and cellular coagulative necrosis in H&E staining was analyzed based on MRI images before RFA treatment (*blue square*), immediately after RFA treatment (*orange square*), and at 28 days (*yellow square*). **(B)** Graph showing the mean tumor volume changes in MRI analysis after with/without RFA treatment in the study groups. **(C)** Graph showing the degree of cellular coagulative necrosis after RFA treatment in study groups. Note *** <0.001.

Follow-up MRI images of the sine and square electrical waveforms are shown in Figures 5A. Tumor growth was significantly different between the groups during MRI follow-up (*p* < 0.001). Furthermore, the tumor growth inhibitory effect in the square group (135.64% ± 19.83%; *p* < 0.001) was higher at 28 days of MRI follow-up than that in the sine group (208.98% ± 21.15%) (Figure 5B). A relatively low-intensity signal within the tumor was observed on T2-weighted MRI immediately after RFA on follow-up MRI images. Relatively lower-intensity signals of the tumor in the square group were observed compared to those in the sine group immediately after RFA. In addition, extensive lower-intensity signals of tumors in the square group were observed compared to those in the sine group. A correlation between coagulative necrosis on histopathology and lower-intensity signals on MRI images was observed. Coagulative necrosis throughout the tumor was consistently observed on H&E examination at immediately after RFA. However, the degree of cellular coagulative necrosis was significantly higher in the square group (3.75 ± 0.68; *p* < 0.001) than in the sine group (3.06 ± 0.66) immediately after RFA. The degree of cellular coagulative necrosis decreased at 28 days but was significantly higher in the square group (3.13 ± 0.62; *p* < 0.001) than in the sine group (2.31 ± 0.79) (Figure 5C).

### Histological findings

**FIGURE 6:**
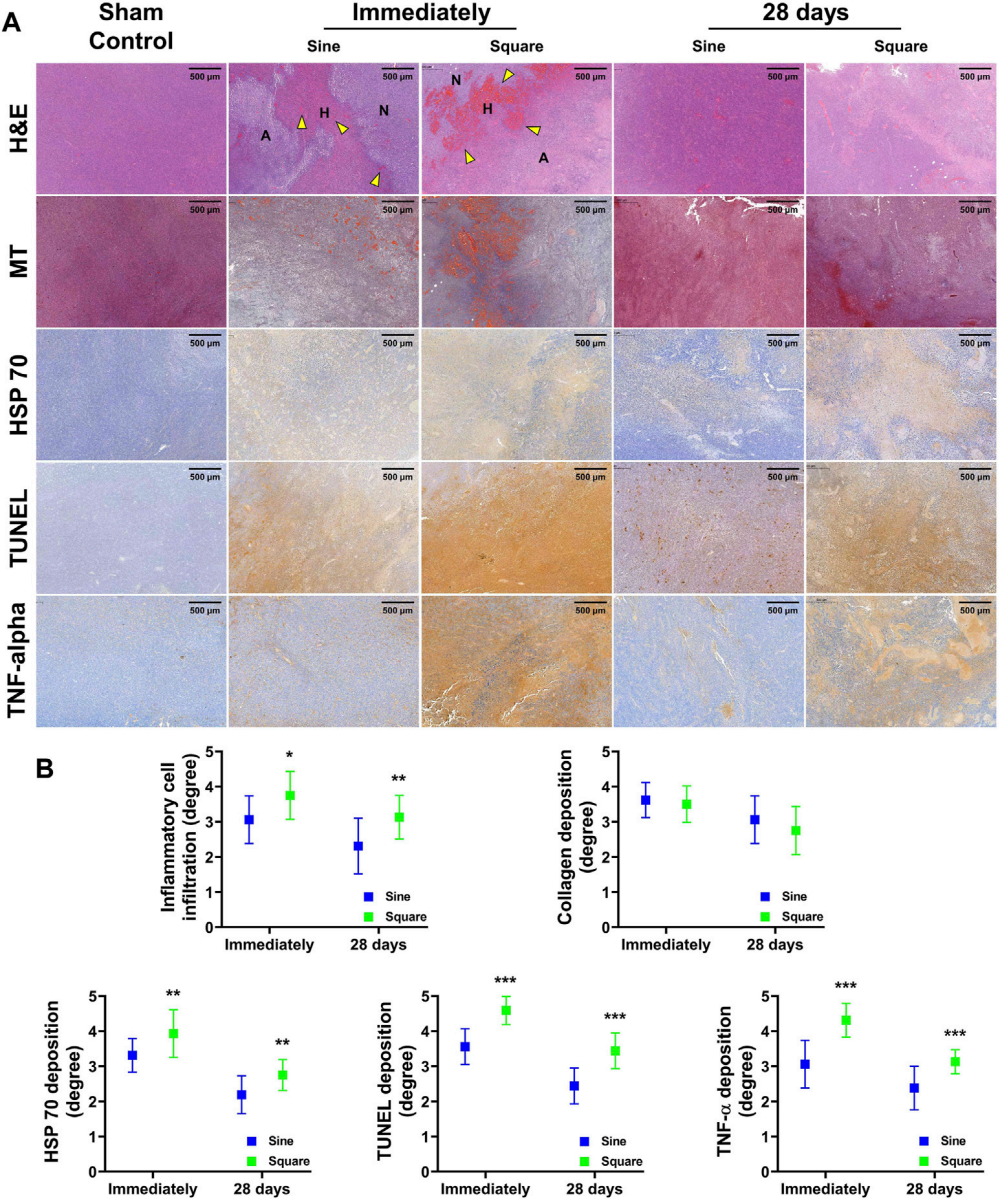
*In-vivo* histological findings after radiofrequency ablation (RFA) in the mice tumor model. **(A)** Representative histological and immunohistochemical images of all groups. The ablation zone with coagulated red blood cells (*yellow arrowheads*) was shown after RFA treatment. **(B)** Serial histopathological findings for the mice tumor model immediately after RFA and 28 days. Note. Ablated, A; Hemorrhage, H; Normal, N; Hematoxylin and eosin, H&E; Masson’s trichrome, MT; heat shock protein 70, HSP 70; terminal deoxynucleotidyl transferase-mediated dUTP nick and labeling, TUNEL; Tumor necrosis factor-alpha, TNF-α. * <0.05, ** <0.01, *** < 0.001.

The histological findings are summarized in Supplementary Table S3 and shown in Figure 6. The degree of inflammatory cell infiltration, degree of HSP 70 deposition, degree of TUNEL deposition, and degree of TNF-α deposition were significantly higher in the square group than in the sine group immediately after RFA (all variables; *p* < 0.05). The degree of inflammatory cell infiltration, degree of HSP 70 deposition, degree of TUNEL deposition, and degree of TNF-α deposition were decreased at 28 days compared with immediately after RFA. Consistently, the degree of inflammatory cell infiltration, degree of HSP 70 deposition, degree of TUNEL deposition, and degree of TNF-α deposition were significantly higher in the square group than in the sine group at 28 days (all variables; *p* < 0.01). However, there was no significant difference between the groups in the degree of collagen deposition immediately (*p* = 0.992) or 28 days (*p* = 0.327). Moreover, a hemorrhagic rim was observed in the RFA-treated group immediately after RFA. An extensive and large hemorrhagic rim was observed in the square group immediately after RFA compared with sine group.

## Discussion

The results of this study demonstrated that RFA with a square electrical waveform could provide a wider ablation area, and ablated margins with a spherical shape became clearer compared to RFA with at the sine electrical wave under the same RF parameters in the porcine liver. MRI findings confirmed that tumor growth in the square group was significantly suppressed compared to that in the sham control and sine groups in the mouse tumor model. Additionally, histological findings revealed extensive coagulative necrosis with protein denaturation and congestion in the central ablation area of observed in the square group. Consistently, necrotic tumor cells and heat shock proteins with coagulative necrotic cells were observed throughout the ablated area in the square group compared to the sine group.

RFA has been confirmed to be promising for minimally invasive local tumor treatment with low complications over the past decade (Krupka et al., 2006). However, the success of RFA treatment is limited to relatively small tumors less than 3 cm (Solbiati et al., 2001). Various preclinical studies have been conducted to enhance therapeutic outcomes and expand the ablation ranges according to changes in the RFA-related variables such as electrical energy, energy application time, and temperature (Petersen et al., 2000; Itoi et al., 2012; Won et al., 2023). Conventionally, the ablation range was controlled by adjusting the electrical energy output applied to biological tissue, but as the impedance of the path through which the current flows increased, the ablation range was limited (Bourier et al., 2020; Bhaskaran et al., 2016). In addition, the ablation range increased by adjusting the time for applying RF energy, but there was little change after the application time of 8 min (Goldberg et al., 1995; Patterson et al., 1998). Furthermore, if RF energy is applied for a long time at a high temperature >100°C, water evaporation leads to the tissue charring. Subsequently, impedance increased dramatically, followed by the “roll-off” effect which is a limited RF energy transfer (Zhang et al., 2015).

In this study, an RFA generator capable of switching electrical waveforms was developed, and the tissue ablation ranges according to the two types of electrical waveforms under same the RF parameters were investigated. Square electrical waveforms have the advantage of enabling precise temperature control. Square electrical waveforms have a higher ripple frequency and smaller ripple amplitude in temperature compared to sine electrical waveforms. The temperature ripple frequency of the sine and square electrical wave were approximately 0.08 Hz and 0.14 Hz, respectively. Sine electrical waveforms cause fluctuations in energy output over time, leading to variations in current and thermal. In contrast, square electrical waveforms with many harmonics and consistent current flow ensure more heat generation and steady temperature, respectively (Sonerud et al., 2009; Li et al., 2020). This can result in a broader ablation range and the formation of clear ablation boundaries (Bao and Mazumder, 2022). The previous study also demonstrated that the square electrical waveform delivers more energy and achieves a larger ablation area in all cases compared to the sine electrical waveform even using half-sine and half-square waveform (Zhang et al., 2015). Even when the same current and thermal energy are applied, a higher ripple frequency in temperature allows heat to be distributed more evenly (Zhang et al., 2015). This ensures that excessive heat is not concentrated at a specific point but is instead spread evenly over a wide tissue area. Consequently, the use of square electrical waveforms forms a large ablation zone with clear boundaries. To achieve temperature control in the RF generator, the same RF parameters were applied to sine or square electrical waveforms, both *in-vitro* and *in-vivo*. However, as shown in Figure 4A, the square electrical waveform consistently demonstrated a smaller fluctuation range with respect to the temperature change compared with the sine electrical waveform. Additionally, it is well-known that tumor tissue has lower impedance than healthy tissue (Haemmerich et al., 2009). Also, previous studies have shown that the square electrical waveform can make lower impedance compared to the sine electrical waveform (Zhang et al., 2015). Lower impedance allows higher current to flow at the same power, which is proportional to the generated thermal energy (Won et al., 2022). These observations mean that using the square electrical waveform could achieve a well-defined boundary and a larger ablation size. Furthermore, previous studies using finite element modeling have demonstrated the superior ablation area achieved by the square electrical waveform; however, these studies did not consider for the specific environment of tumor tissue (Lim et al., 2010; Zhang et al., 2015). The improved ablation area of the square electrical waveform obtained from our model incorporating the characteristics of tumor tissue more accurately reflects the clinical environment. Therefore, our current findings support that RFA with the square electrical waveform seems to be an effective therapeutic option for treating tumor ablation compared to conventional RFA with a sine electrical waveform in a mouse tumor model.

The use of follow-up MRI images after RFA application confirms successful treatment of the lesion at an early stage (Boaz et al., 1998; Nour and Lewin, 2005). In this study, low-intensity signals on T2WI were observed throughout the tumor immediately after RFA treatment in the square group. The low signal on T2WI indicates complete thermal damage immediately after RFA treatment (Nour and Lewin, 2005). Changes in MRI signal intensity may be due to complicated reasons, including the combined effects of cell lysis and protein denaturation after RFA (Lee et al., 2001). Consistently, histopathology findings demonstrated that extensive cellular coagulate necrosis within the tumor was observed immediately after RFA treatment, demonstrating superior tumor management at 28 days in the square group, suggesting that the square electrical waveform resulted in a higher intensity of RF energy delivery, which may have resulted in more extensive cellular coagulation necrosis in the square group. Histopathological examination of the marginal area revealed an increased hemorrhagic rim with sinusoid congestion and red blood cell accumulation with neoplastic cell degeneration. This area is consistent with previous reports that it corresponds to the transition zone from internal coagulative necrosis to external normal tumor tissue (Lee et al., 2001). Rai et al. (2005) reported that techniques to induce apoptosis and increase the size of the transition zone would further increase the efficacy of RFA in clinical practice. In addition, in the square group, degeneration of heat shock protein and necrosis with more coagulated blood cells were observed immediately after RFA, and tumor cells that had not fully recovered were observed after 28 days. Therefore, the square group seemed to have a superior survival rate and a lower residual growth rate than the sine group because of the increased transition zone and extensive cellular coagulative necrosis in tumor management.

Various commercially available electrodes have been designed and developed to mechanically overcome the extent of the ablation zone (Ni et al., 2005). These electrodes include bipolar electrodes, internally cooled electrodes, multiple and expandable electrodes with an enlarged electric field, and wet electrodes with saline perfusion through the electrode into the tissue. These have been demonstrated to create larger ablation volumes than conventional single RFA electrodes (Ni et al., 2005; Lee et al., 2012). The current study was conducted using monopolar electrodes both *ex-vivo* and *in-vivo*. The combination of optimal electrical RFA variables and an optimal mechanical RFA electrode may result in effective RFA treatment. Therefore, future research on the combination of square electrical waveforms and multi-functional electrodes is needed to determine the optimal clinical efficacy of RFA.

This study has several limitations. First, only one cancer cell line and subcutaneous model were used. Further use of other cancer cell lines and orthotopic models is needed to characterize the influence of different tumor microenvironments. Second, only a few representative markers of hyperthermia- and necrosis-related RFA were evaluated by pathological staining in this study. Additional immunofluorescence and IHC staining may aid in the quantitative assessment of the microenvironment, including angiogenesis and lymphangiogenesis. Third, it is necessary to confirm changes in the tumor tissue depending on the time point after the RFA application to ensure the safety of the application. Fourth, this study did not consider the heat sink effect around large blood vessels. Convective heat transfer via blood flow in a large blood vessel acts as a heat sink. Large animal models with tumors and convective heat transfer analyses must be considered together to validate to the tumors coexisting with large blood vessels. Fifth, although the square electrical waveform achieved enlarged ablation area in a mouse tumor model, additional study was required to confirm the efficacy and safety of RFA using the square electrical waveform in human tumor microenvironment for further translational research. Nevertheless, the current study seems to be a promising approach for the enhanced ablation ranges of RFA with a square electrical waveform for tumor tissue and indicates its potential for human applications.

In conclusion, RFA with square electrical waveforms demonstrated a spherical ablated shape and wider ablation range in the porcine liver. RFA using square electrical waveforms has been demonstrated to inhibit tumor growth and results in higher survival rates owing to intensive neoplastic cell degeneration and coagulative necrotic cells in the tumor. Additionally, an enhanced ablation range was achieved by adjusting the RF electrical waveform parameters to consistently deliver a higher intensity of RF energy output to the target tissue, resulting in extensive cellular coagulate necrosis. Although further studies are needed to verify its efficacy in a large animal tumor model, RFA with square electrical waveforms has therapeutic potential for tumor suppression, with an enhanced ablation range and extensive cellular coagulation necrosis.

## Data Availability

The original contributions presented in the study are included in the article/Supplementary Material, further inquiries can be directed to the corresponding authors.
