# Supplementary material for: Radiofrequency ablation with sine and square electrical waveforms to enhance ablation range
Source: Front Bioeng Biotechnol. 2024 Aug 21;12:1450331. doi: 10.3389/fbioe.2024.1450331 (PMC11372458; doi:10.3389/fbioe.2024.1450331)
Supplement: Supplementary file 1 [file DataSheet1.docx]

SUPPLEMENTARY MATERIALS

**Radiofrequency Ablation with Sine and Square Electrical Waveforms to Enhance Ablation Ranges**

*Dong-Sung Won^1a^, Jinsu An^2a^, Ji Won Kim^1,3^, Yubeen Park^1,3^, Sang Soo Lee^3^,* *Hyoung-Sik Kim^4b^, and Jung-Hoon Park^1,5b^*

*^1^Biomedical Engineering Research Center, Asan Institute for Life Sciences, Asan Medical Center, 88 Olympic-ro 43-gil, Songpa-gu, Seoul, 05505, Republic of Korea*

*^2^Department of Biomedical Engineering, School of ICT Convergence Engineering, College of Science & Technology, Konkuk University, 268 Chungwon-daero, Chungju-si, Chungcheongbuk-do 27478, Republic of Korea*

*^3^Department of Gastroenterology, Asan Medical Center, University of Ulsan College of Medicine, 88 Olympic-ro 43-gil, Songpa-gu, Seoul 05505, Republic of Korea*

*^4^Department of Mechatronics Engineering, School of ICT Convergence Engineering, College of Science & Technology, Konkuk University, 268 Chungwon-daero, Chungju-si, Chungcheongbuk-do 27478, Republic of Korea*

*^5^Department of Convergence Medicine, Asan Medical Center, University of Ulsan College of Medicine, 88 Olympic-ro 43-gil, Songpa-gu, Seoul, 05505, Republic of Korea*

^a^ D.-S.W. and J.A. contributed equally to this work and are co-first authors.

^b^ H.-S.K. and J.-H.P. contributed equally to this work and are co-corresponding authors.

**Correspondence:**

Hyung-Sik Kim, Ph.D.

Department of Mechatronics Engineering, School of ICT Convergence Engineering, College of Science & Technology, Konkuk University, 268, Chungwon-daero, Chungju-si, Chungcheongbuk-do 27478, Republic of Korea

Tel: 82-43-840-3767

E-mail: [hskim98@kku.ac.kr](mailto:hskim98@kku.ac.kr)

Jung-Hoon Park, Ph.D.

Department of Convergence Medicine, Asan Medical Center, University of Ulsan College of Medicine, 88 Olympic-ro 43-gil, Songpa-gu, Seoul, 05505, Republic of Korea

Biomedical Engineering Research Center, Asan Institute for Life Sciences, Asan Medical Center, 88 Olympic-ro 43-gil, Songpa-gu, Seoul, 05505, Republic of Korea

Tel: 82-2-3010-4123 Fax: 82-2-476-0090

E-mail: [jhparkz@amc.seoul.kr](mailto:jhparkz@amc.seoul.kr)


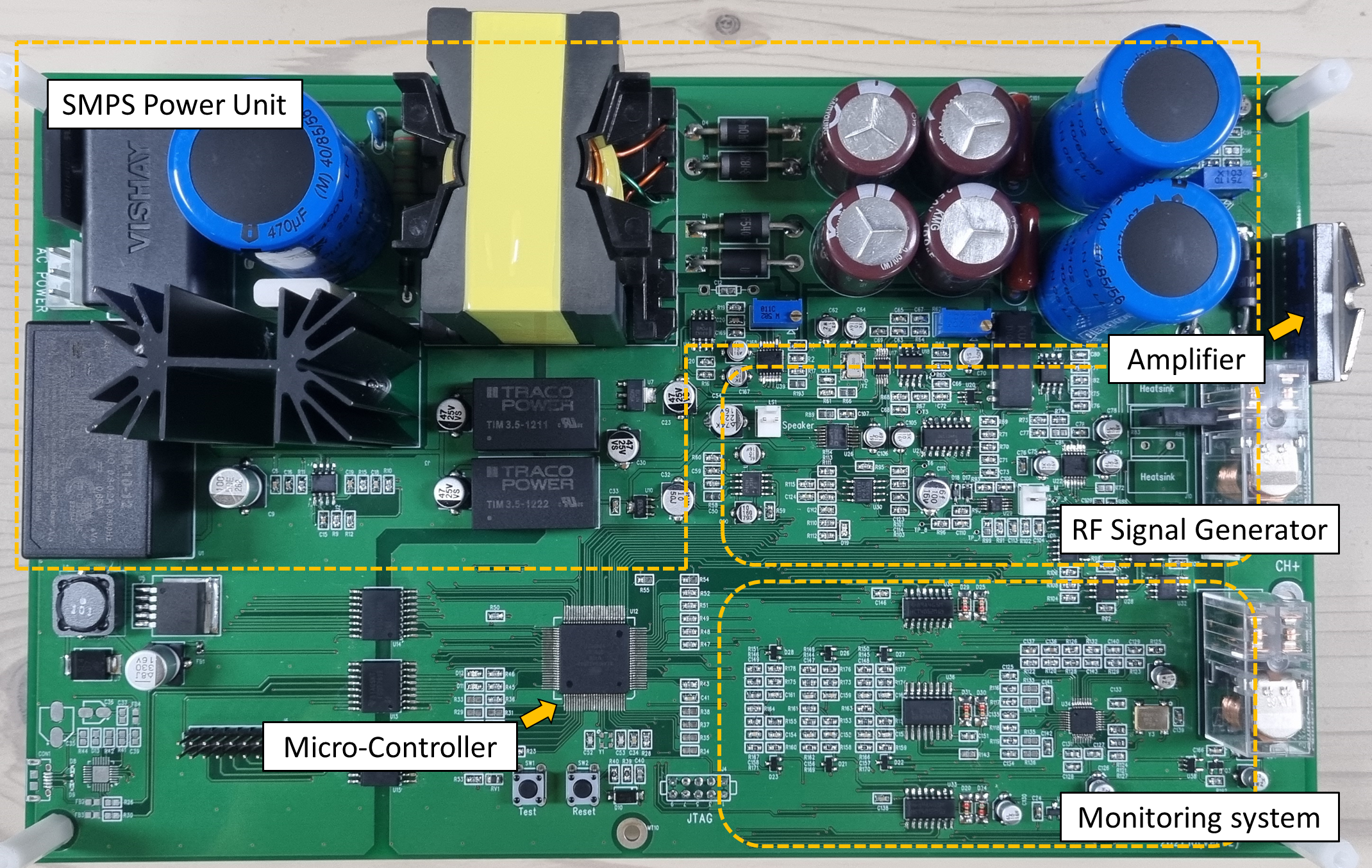


**Supplementary Figure 1.** Printed circuit board of the RF generator.


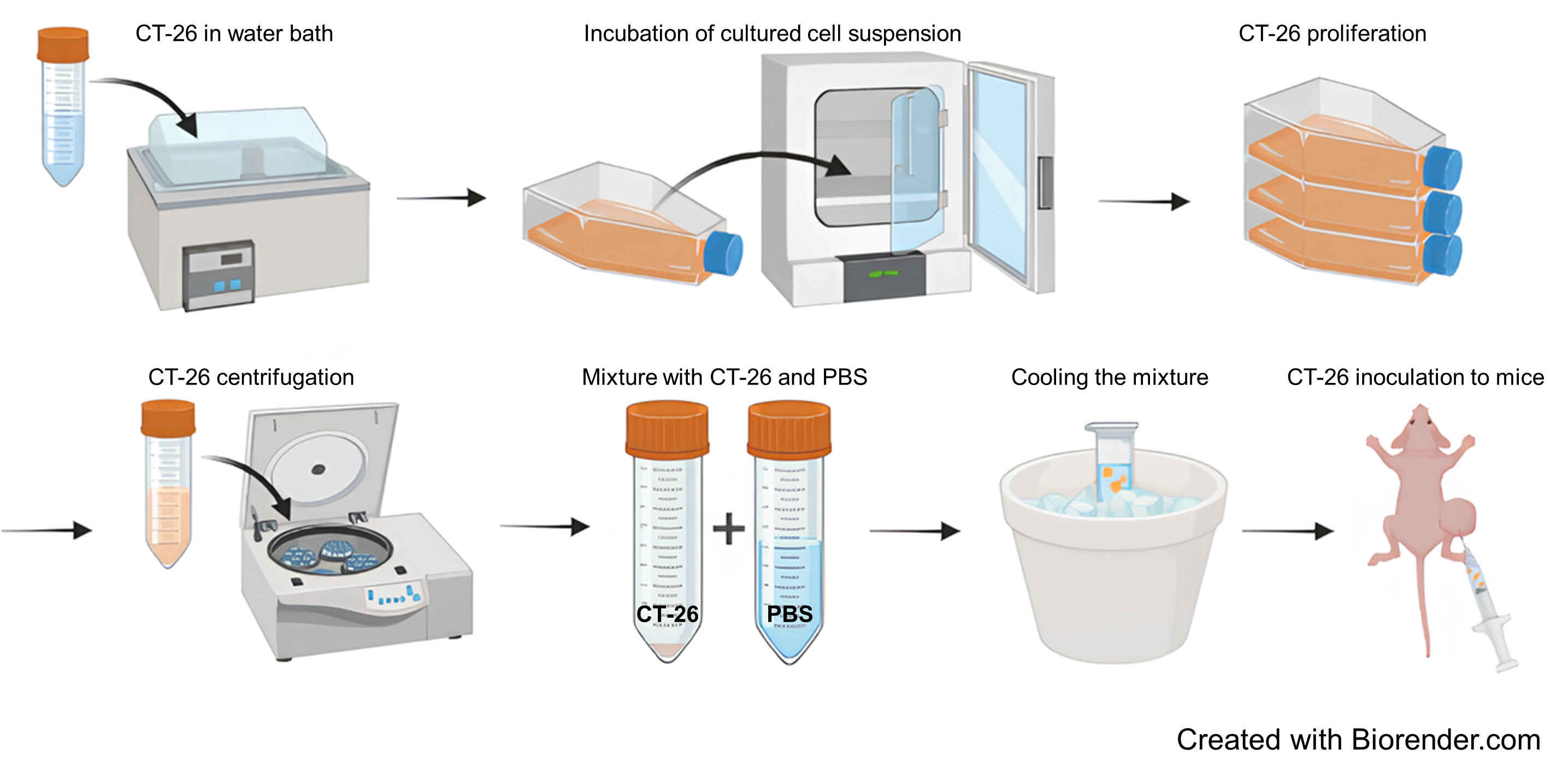


**Supplementary Figure 2.** Schematic illustration of the cell culture for creation of the mouse tumor model.


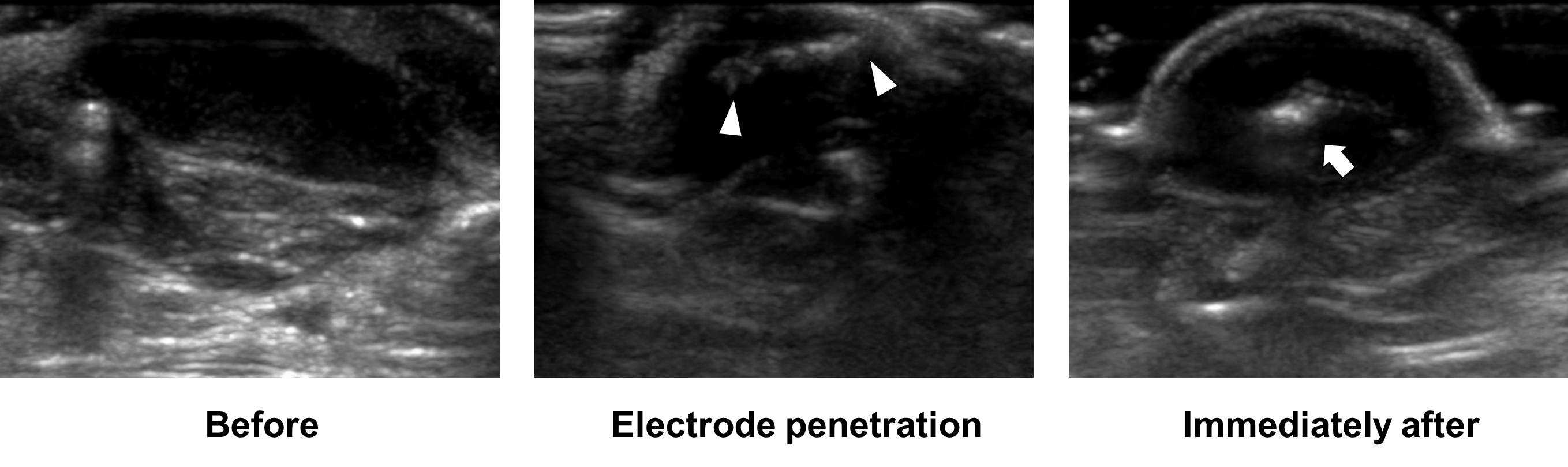


**Supplementary Figure 3.** Representative ultrasonographic images showing the technical steps of RFA treatment using monopolar needle electrodes. The electrode (*arrowheads*) penetrates the center of the tumor under ultrasonographic guidance and hyperechogenicity (*arrows*) is observed immediately after RFA procedure at the tumor center in the square group.

**Supplementary Table 1.** The parameters of developed RF generator

|  | **Control Parameter** |
| --- | --- |
| Waveform | Sine and Square wave |
| Frequency | 10 kHz ~ 500 kHz **/** 1 kHz |
| Power | 5 Watt ~ 60 Watt **/** 5 Watt |
| Temperature | 50℃ ~ 100℃ **/** 5℃ |
| Impedance measurement | 10 Ω ~ 1.5 kΩ |
| Operating Time | 5 Sec ~ 1800 Sec / 5 Sec |

**Supplementary Table 2.** Characteristic of the sine or square electrical waveform using testing resistive load.

|  | **Sine Wave** | | | **Square Wave** | | |
| --- | --- | --- | --- | --- | --- | --- |
|  | Voltage(V) | Current(mA) | Power(W) | Voltage(V) | Current(mA) | Power(W) |
| 5 W | 21.20 | 516 | 5.47 | 14.60 | 342 | 4.99 |
| 10 W | 29.20 | 681 | 9.94 | 20.80 | 504 | 10.48 |
| 15 W | 36.10 | 854 | 15.41 | 25.21 | 607 | 15.30 |
| 20 W | 41.82 | 944 | 19.74 | 29.62 | 673 | 19.93 |

**Supplementary Table 3.** *In-vivo* experimental findings after radiofrequency ablation with sine or square electrical waveform.

|  | **Sine Wave** | | **Square Wave** | | ****p*-value** | |
| --- | --- | --- | --- | --- | --- | --- |
|  | Immediately  (a) | 28 days  (A) | Immediately  (b) | 28 days  (B) | a vs. b | A vs. B |
| Cellular coagulative necrosis (degree) | 2.87 ± 0.50 | 2.12 ± 0.88 | 3.81 ± 0.54 | 3.12 ± 0.71 | .001 | .001 |
| Inflammatory cell infiltration (degree) | 3.06 ± 0.68 | 2.31 ± 0.79 | 3.75 ± 0.68 | 3.13 ± 0.62 | .025 | .006 |
| Collagen deposition  (degree) | 3.62 ± 0.50 | 3.06 ± 0.68 | 3.50 ± 0.52 | 2.75 ± 0.68 | .992 | .327 |
| HSP 70 deposition  (degree) | 3.31 ± 0.48 | 2.19 ± 0.54 | 3.93 ± 0.68 | 2.75 ± 0.44 | .004 | .010 |
| TUNEL deposition  (degree) | 3.56 ± 0.51 | 2.44 ± 0.51 | 4.59 ± 0.40 | 3.44 ± 0.51 | .001 | .001 |
| TNF-α deposition  (degree) | 3.06 ± 0.68 | 2.38 ± 0.62 | 4.31 ± 0.48 | 3.13 ± 0.34 | .001 | .001 |

Note. Data are represented as mean ± standard deviation. * Student’s *t-test*. HSP 70: heat shock protein 70, TUNEL: terminal deoxynucleotidyl transferase-mediated dUTP, and TNF-α: tumor necrosis factor-α.
